# First-Principles Studies of Hydrogen Adsorption at Pd-SiO_2_ Interfaces

**DOI:** 10.3390/s150614757

**Published:** 2015-06-22

**Authors:** Yoshihiro Irokawa, Mamoru Usami

**Affiliations:** 1 National Institute for Materials Science, 1-1 Namiki, Tsukuba 305-0044, Japan; 2 ASMS Co., Ltd., 1-10-7 Higashi-Gotanda, Shinagawa, Tokyo 141-0022, Japan; E-Mail: usami@asms.co.jp

**Keywords:** hydrogen, interfaces, dielectric

## Abstract

The interaction of hydrogen with Pd-SiO_2_ interfaces has been investigated for the first time using first-principles calculations based on density functional theory. The hydrogen-induced polarization at the Pd-SiO_2_ interfaces was evaluated using Pd-SiO_2_ interface supercells. As a result, the potential change induced by interfacial hydrogen atoms was not observed even for hydrogen concentration of ~1.3 × 10^15^ cm^−2^ at the Pd-SiO_2_ interface. This result implies that hydrogen does not create an electric double layer at the Pd-SiO_2_ interface but change the property of the SiO_2_ region, resulting in the hydrogen sensitivity of the devices.

## 1. Introduction

Hydrogen is expected to be one of the alternative energy resources of the future, and hydrogen sensors are some of the most important components in a hydrogen-based society. The interaction of hydrogen with semiconductor devices has long been studied for applications in various semiconductor-based hydrogen sensors, particularly for the detection of leaks in various hydrogen systems, including space- and ground-based vehicles and hydrogen production processes [1]. Intensive research has led to a model which attributes the reaction mechanism of the devices to hydrogen to the formation of a hydrogen-induced dipole layer at the metal-dielectric interface [2]. Molecular hydrogen adsorbs on Pd or Pt surface and dissociates. Hydrogen atoms diffuse through Pd or Pt and adsorb at the metal-oxide interface, forming a dipole layer. The dipole layer is responsible for the work function change, for example, showing up as a voltage shift of the capacitance-voltage (*C-V*) characteristics of the device. Despite the existence of a considerable quantity of experimental data, however, there are still some debates as to the origin of the hydrogen sensitivity. For instance, there is a possibility that the hydrogen sensitivity of the devices results from the change of the catalytic metal property just as observed in devices like Kelvin probes. As for the change in Pd work function, however, previous literatures report that the work function decreases at low temperatures like 78 K but increases above 120 K under ultrahigh vacuum conditions (UHV) [3,4,5]. The changes in the work function varies depending on the Pd structure and plane direction, but the work function increases unless at quite low temperatures. On the other hand, it is reported that voltage shifts in *C-V* characteristics in Pd-metal oxide semiconductor (MOS) structure on p-Si show negative values in the presence of hydrogen at 473 K [2], suggesting that the Pd work function apparently decreases. Similar voltage shifts of the *C-V* characteristics in Pd-MOS structure upon hydrogen exposure were observed even at 293 K [6]. These results seem not to be consistent with literatures which report that the Pd work function increases unless at quite low temperatures, implying that the change in the Pd work function is not the dominant factor for hydrogen sensitivity. Once Pd is incorporated into device structures as the electrodes, it is difficult to distinguish the change of the Pd work function per se, because the change in the semiconductor material property, including dielectrics and semiconductor layers, would be apparently reflected in measured changes in the work function. Therefore, it is important to investigate the hydrogen response mechanism of Pd-MOS devices in order not only to improve the hydrogen sensors but also to secure the reliability of electronic devices. As another example which shows that the change in the catalytic metal work function is not the dominant factor for hydrogen sensitivity, there is a literature which reports hydrogen responses of MIS Pt-GaN diodes with both SiO_2_ and Si_x_N_y_ dielectrics [7]. In the report, the characterized devices are completely identical structure except for the dielectrics between the Pt and the GaN. However, the hydrogen responses are totally different. For a MIS Pt-GaN diode with a SiO_2_ dielectric, the *C-V* curve in hydrogen significantly shifts toward negative bias values. In sharp contrast, for a MIS Pt-GaN diode with a Si_x_N_y_ dielectric, the *C-V* curve does not show any shifts upon exposure to hydrogen. This result indicates that the change in the catalytic metal work function is not the dominant factor for sensitivity, because these two devices have the same Pt metal as the contacts, and one shows hydrogen response, but another does not show any response to hydrogen.

The computational study of the electronic states using first-principles calculations based on density functional theory (DFT) recently plays a critical role in a large variety of electronic materials. As for the interaction of hydrogen with Pd-SiO_2_ interfaces, however, no literature has been published so far. The first-principles calculations based on DFT are applied to obtain hetero-interface band offsets and would also be a powerful tool in order to evaluate the hydrogen-induced polarization at the metal-dielectric interface.

In this report, the hydrogen-induced polarization at the Pd-SiO_2_ interfaces was evaluated using Pd-SiO_2_ interface supercells incorporated with hydrogen. As a result, the potential change induced by interfacial hydrogen atoms was not observed even for hydrogen concentration of ~1.3 × 10^15^ cm^−2^ at the Pd-SiO_2_ interface. The obtained results imply that hydrogen does not create an electric double layer at the Pd-SiO_2_ interface but change the property of the SiO_2_ region.

## 2. Experimental Section

Previous literatures show that the Pd-SiO_2_ interfaces are responsible for hydrogen sensitivity of the Pd-SiO_2_-Si structure [2], and in this study, Pd-SiO_2_ interface supercells are utilized in order to evaluate the hydrogen-induced polarization at the Pd-SiO_2_ interfaces.

Our first-principles methods are based on DFT within the general gradient approximation (GGA). The calculations were performed using the PHASE/0 package [8], which is based on plane-wave ultrasoft pseudopotentials. The wavefunctions were expanded in the plane waves up to the kinetic energy cutoff of 340 eV. Brillouin-zone integrations for Pd-SiO_2_ interface supercells were carried out using a 2 × 2 × 1 Monkhorst-Pack **k**-point grid. All structural models were fully relaxed until the atomic forces were less than 2.5 × 10^−2^ eV/Å. The total number of atoms in this supercell model was 171, including 24 adsorbed hydrogen atoms at the both Pd-SiO_2_ interfaces.

**Figure 1:**
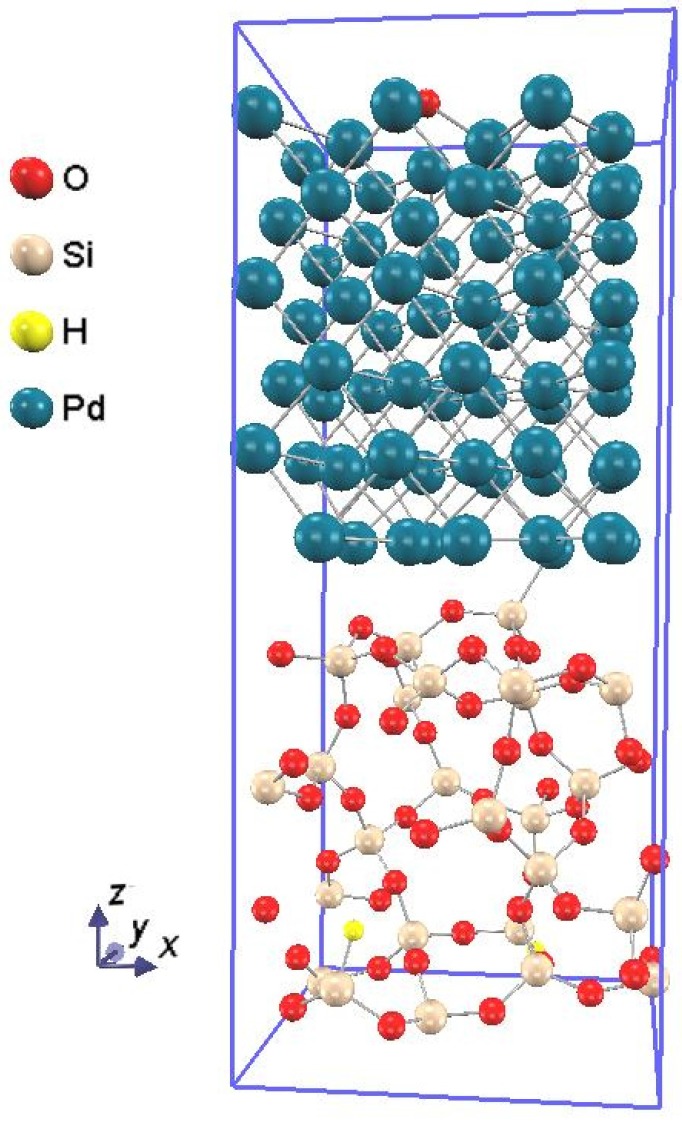
The atomic structure of the Pd-SiO_2_ interface supercell. O, Si, H, and Pd atoms are depicted by the red, flesh color, yellow, and dull blue spheres, respectively.

Figure 1 shows the atomic structure of the Pd-SiO_2_ interface supercell, and the procedure to construct the model is described in the following sequence: First, in order to model the Pd-SiO_2_ interfaces, amorphous SiO_2_ structures were generated using molecular dynamics code, LAMMPS [9]. The lattice parameters of the configured SiO_2_ supercell were *a* = 10.28 Å, *b* = 8.90 Å, *c* = 11.31 Å, respectively. Then, the lattice constants and all the atoms were allowed to relax to minimize the total energy of the supercell. A vacuum layer was attached to the relaxed SiO_2_ supercell, and the SiO_2_ structure were optimized to minimize the total energy of the supercell, introducing hydrogen termination to the dangling bonds.

**Figure 2:**
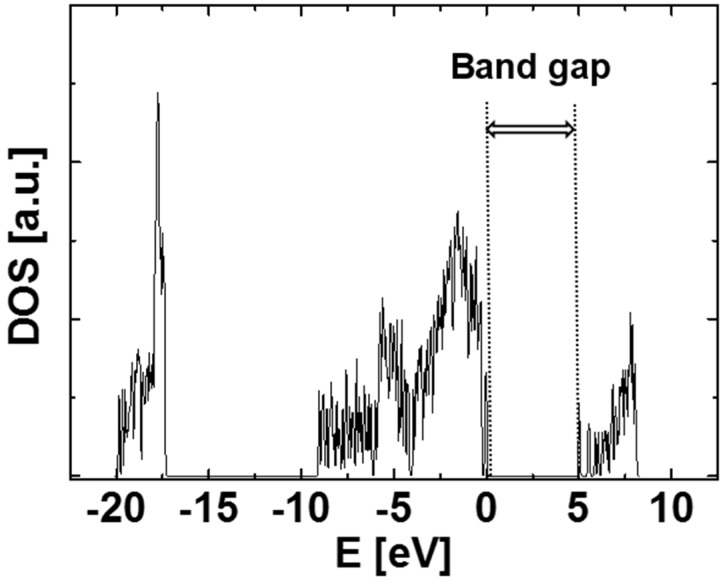
The calculated density of states (DOS) of the generated SiO_2_ structure.

Figure 2 shows the calculated density of states (DOS) of the generated SiO_2_ structure. The band gap from the calculation shown in this figure is ~5.1 eV. Note that GGA typically underestimates the band gap. The spectrum shown in Figure 2 is similar to that of bulk quartz, which was calculated using the VASP code [10]. The literature band gap of SiO_2_ is 5.9 eV, although our calculation shows it is 5.1 eV. The difference in the values of band gap may result from the initially hypothesized SiO_2_ structure, *i.e.*, we calculate amorphous SiO_2_ structure, but bulk quartz is used in the literature.

Second, Pd-SiO_2_ contacts were formed. For fcc metal, Pd, whose lattice constant is 3.89 Å, the area of (100) plane is 3.89 Å × 3.89 Å. Considering the Pd-SiO_2_ interface, for the area of 6 times Pd(100) plane, the in-plane lattice mismatch with the configured SiO_2_ supercell is 0.81%. In order to form Pd-SiO_2_ contacts, six layers of Pd are included in the fabricated SiO_2_ supercell, and the lattice constant along the interface normal, z-axis, and all the atoms were allowed to relax to minimize the total energy of the supercell, resulting in the lattice constant, *c* = 25.40 Å.

The incorporation energy of 2*n* hydrogen atoms at the both Pd-SiO_2_ interfaces, *E*_2*n*H_, was computed, after the structural optimization, using

(1) $$E_{2n\text{H}}=E(\text{Pd}/\text{Si}O_{2}+2n\text{H})-(E(\text{Pd}/\text{Si}O_{2}+2(n-1)\text{H})+E({\text{H}}_{2}))$$

where *n* is natural number, *E*(Pd/SiO_2_+2*n*H) is the energy of the Pd-SiO_2_ interface supercell with 2*n* hydrogen atoms incorporated at the both Pd-SiO_2_ interfaces, *E*(Pd/SiO_2_) is the energy of the Pd-SiO_2_ interface supercell, and *E*(H_2_) is the energy of a H_2_ molecule.

## 3. Results and Discussion

**Figure 3:**
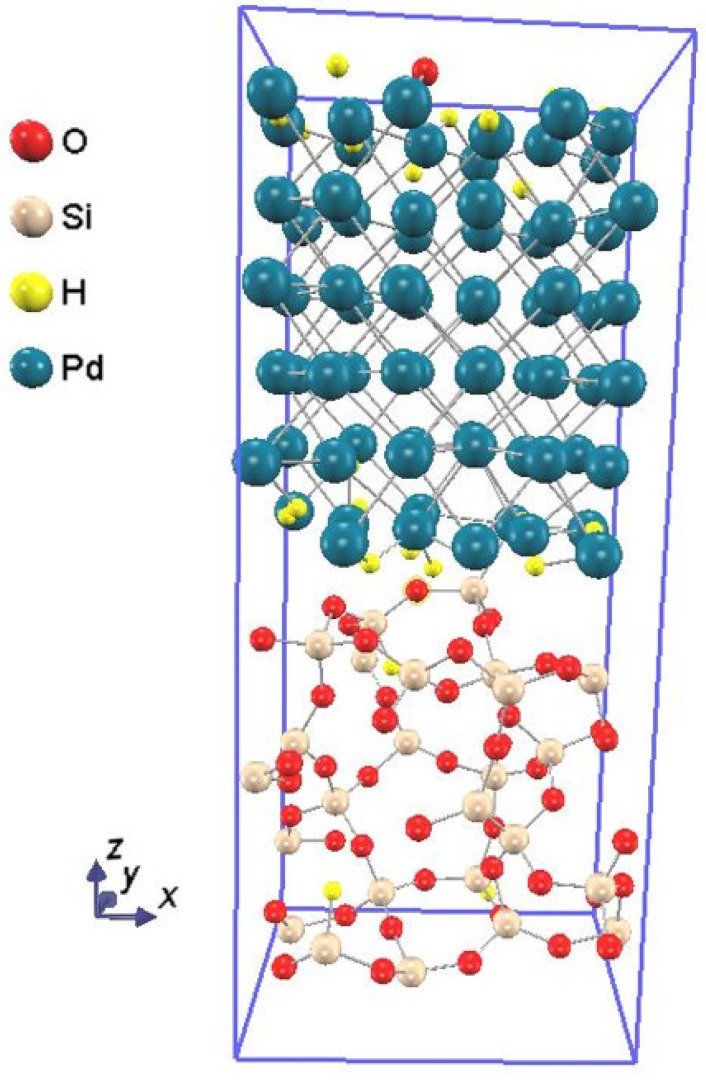
The Pd-SiO_2_ interface supercell with 24 hydrogen atoms adsorbed at both Pd-SiO_2_ interfaces of the supercell, which corresponds to a hydrogen concentration of ~1.3 × 10^15^ cm^−2^ at the Pd-SiO_2_ interface.

The hydrogen sensitivity of Pd-SiO_2_-Si structures was studied experimentally, and the hydrogen concentration at the Pd-SiO_2_ interface was estimated to be ~1 × 10^14^ cm*^−^*^2^ using the hydrogen adsorption isotherm analyses and *C-V* characteristics, when the *C-V* curve showed a shift of ~0.8 V [2]. This hydrogen concentration corresponds to ~1 hydrogen atom at the Pd-SiO_2_ interface of the constructed supercell. On the other hand, elastic recoil detection (ERD) measurements were performed to investigate hydrogen response mechanism of Pt-GaN Schottky diodes, and quantitative analysis revealed a hydrogen concentration of ~1.3 × 10^15^ cm^−2^ at the Pt-GaN interface [11], which corresponds to ~12 hydrogen atoms at the Pd-SiO_2_ interface of the supercell. In this study, a maximum number of 24 hydrogen atoms was randomly inserted two-by-two into both Pd-SiO_2_ interfaces of the supercell. After the structural optimization was performed, the incorporation energy of 2*n* hydrogen atoms at the Pd-SiO_2_ interfaces, *E*_2*n*H_, was computed. If the *E*_2*n*H_ is negative, 2*n* hydrogen atoms are stably incorporated at the Pd-SiO_2_ interfaces. As a result, the *E*_24H_ was found to be negative, meaning that a hydrogen concentration of ~1.3 × 10^15^ cm^−2^ at the Pd-SiO_2_ interface is stable. Figure 3 shows the Pd-SiO_2_ interface supercell with 24 hydrogen atoms incorporated in both Pd-SiO_2_ interfaces of the supercell. Note that a hydrogen atom inserted into the Pd-SiO_2_ interface tends to adsorb to the side of Pd rather than to that of SiO_2_.

**Figure 4:**
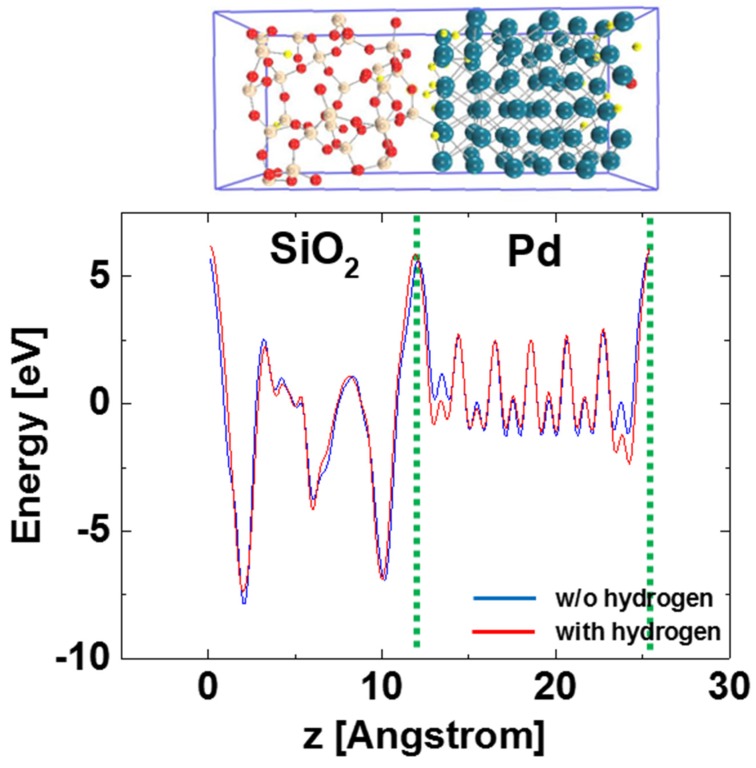
In-plane averaged potential along the interface normal with the two interface planes in the Pd-SiO_2_ interface supercell before and after 24 hydrogen atom adsorption at the Pd-SiO_2_ interfaces.

Figure 4 shows the in-plane averaged potential along the interface normal with the two interface planes in the Pd-SiO_2_ interface supercell before and after 24 hydrogen atom adsorption at the Pd-SiO_2_ interfaces. Here, if hydrogen-induced polarization layers are formed at the Pd-SiO_2_ interfaces, the in-plane averaged potential along the interface normal would shift depending on the amount of the polarization. Similar approach using the averaged potential is found in previous literatures in which hetero-junction interface band offsets are determined using DFT [12]. Note that the averaged potential does not shift after hydrogen adsorption at the Pd-SiO_2_ interfaces as shown in Figure 4, suggesting that hydrogen-induced polarization is not formed at the interfaces. Applied to the case in which 2 hydrogen atoms were adsorbed at the Pd-SiO_2_ interfaces, which corresponds to the hydrogen concentration at the Pd-SiO_2_ interface of ~1 × 10^14^ cm^−2^, no potential shift was also observed. Note that the potential of the Pd locally shows different values along the hydrogen absorption, as shown in Figure 4. This reflects hydrogen atoms per se when calculating in-plane averaged potential and does not show the work function change of the Pd.

**Figure 5:**
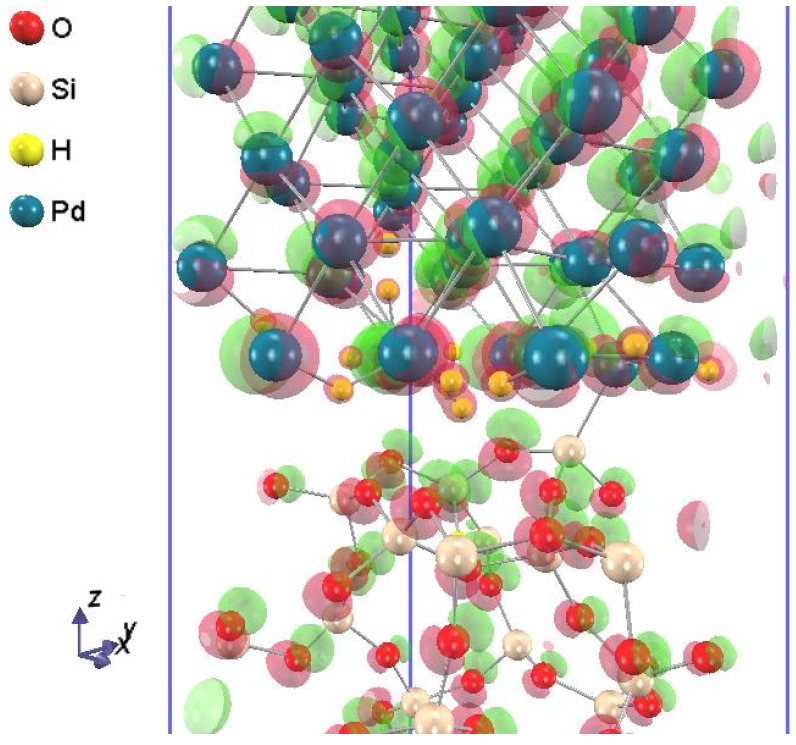
A charge-density difference plot of the Pd-SiO_2_ interface supercell before and after 24 hydrogen atom adsorption at the Pd-SiO_2_ interfaces. Charge accumulation/depletion regions are displayed in translucent red/green isosurface value by ± 0.1 e/Bohr^3^.

In order to elucidate the chemical bonding and charge transfer at the hydrogen adsorbed Pd-SiO_2_ interfaces, a charge-density difference plot of the Pd-SiO_2_ interface supercell before and after 24 hydrogen atom adsorption at the Pd-SiO_2_ interfaces is shown in Figure 5. As shown in Figure 5, a hydrogen atom at the Pd-SiO_2_ interface is surrounded by spherically-distributed electron cloud, and no dipole is observed, again. Note that Pd and O atoms are accompanied by charge accumulation and depletion regions due to the displacement of the atoms after the hydrogen adsorption. These results imply that hydrogen does not create electric double layers at the Pd-SiO_2_ interfaces but change the property of the SiO_2_ region.

Generally speaking, changes in the dielectric layer property upon hydrogen exposure have not drawn much attention. M. C. Petty reported that conduction mechanism in Pd-SiO_2_-Si MIS diodes resulted in Poole-Frenkel emission after exposure to hydrogen [13]. The Pool-Frenkel emission is due to emission of trapped electrons into the conduction band. The supply of electrons from the traps is through thermal excitation. For trap states with Coulomb potentials, the expression is similar to that of the Schottky emission. The barrier height, however, is the depth of the trap potential well. The barrier reduction is larger than in the case of Schottky emission by a factor of 2, since the barrier lowering is twice as large due to the immobility of the positive charge [14]. As a result, the barrier height of Schottky contacts is apparently reduced upon exposure to hydrogen. As for Pt-SiO_2_-GaN MIS diodes, it was reported that exposure of the devices to hydrogen was found to change the conduction mechanisms from Fowler-Nordheim tunneling to Poole-Frenkel emission [7]. According to first-principles calculations of hydrogen-induced defect energy levels in SiO_2_ using a DFT method, K. Xiong *et al*. reported that hydrogen was found to give a deep state in SiO_2_ [15]. This hydrogen-induced change in the band gap state may be related to the origin of the hydrogen sensitivity in Pd-SiO_2_ devices, *i.e.*, the hydrogen-induced defect energy levels in SiO_2_ would be the origin of the Poole-Frenkel emission in Pd or Pt-SiO_2_-GaN MIS diodes after exposure to hydrogen. It is speculated that the calculated DOS of hydrogen-induced defect energy levels in SiO_2_ varies depending on the initially hypothesized SiO_2_ structure.

In addition, if a hydrogen-induced dipole layer is formed at the interface between catalytic metal and semiconductor, we should be able to detect the hydrogen-induced capacitance in the devices. As for an experimental study in order to evaluate the hydrogen-induced electric double layer at the interface between catalytic metal and semiconductor, we reported impedance analysis on hydrogen interaction with Pt-AlGaN/GaN Schottky barrier diodes, revealing that exposure of the diodes to hydrogen did not create an additional RC circuit on the Nyquist plane which represented a hydrogen-induced electric double layer, and the radius of the existing semicircle representing the RC circuit for the semiconductor space charge region was dramatically reduced upon hydrogen exposure [16]. This result indicates that the Schottky barrier height is apparently reduced without forming a hydrogen-induced electric double layer at the interface between the catalytic metal and the semiconductor. Note that a native oxide layer on semiconductor plays a critical role in hydrogen sensing even in metal-semiconductor contacts. O. Weidemann *et al.* reported that an oxidic intermediate layer between the catalytic Schottky contact and the GaN surface was the origin for the hydrogen sensitivity of the devices [17]. It is speculated that the Pt-AlGaN/GaN device has an oxidic intermediate layer between the Pt and the AlGaN layer because the samples was exposed to air before the Pt deposition, and the property of this oxidic layer is changed upon hydrogen exposure, resulting in the apparent reduction of the Schottky barrier height. Therefore, it is assumed that the hydrogen-induced capacitance element is not observed on the Nyquist plane. Note that it is shown that the energy levels of interstitial hydrogen in a wide variety of oxides are calculated, revealing that hydrogen variously alters the band gap state [15].

## 4. Conclusions

In this paper, first-principles calculations were performed to evaluate the hydrogen-induced polarization at the Pd-SiO_2_ interfaces for the first time. As a result, the potential change induced by interfacial hydrogen atoms was not observed even for hydrogen concentration of ~1.3 × 10^15^ cm^−2^ at the Pd-SiO_2_ interfaces. The obtained results imply that hydrogen does not create an electric double layer at the Pd-SiO_2_ interfaces but change the property of the SiO_2_ region, resulting in the hydrogen sensitivity of the devices. Future work will involve more detailed elucidation of the response mechanism of these devices to hydrogen. For example, an investigation using a combinational method both ab initio molecular dynamics (MD) and first-principles calculations would give a more accurate result.
